# LIVING ALONE AND LIKELIHOOD OF HAVING HIGH DEPRESSIVE SYMPTOMS AMONG OLDER ADULTS

**DOI:** 10.1093/geroni/igae098.0617

**Published:** 2024-12-31

**Authors:** Milan Chang (Gudjonsson), Hrafnhildur Eymundsdottir, Sigurveig Sigurdardottir, Lenore Launer, Vilmundur Gudnason, Palmi Jonsson

**Affiliations:** Landspitali University Hospital, Seltjarnarnes, Hofuoborgarsvaoio, Iceland; Landspitali University Hospital, Reykjavik, Hofuoborgarsvaoio, Iceland; University of Iceland, Reykjavik, Hofuoborgarsvaoio, Iceland; National Institutes of Health, Baltimore, Maryland, United States; University of Iceland, Reykjavik, Hofuoborgarsvaoio, Iceland; University of Iceland, Reykjavik, Hofuoborgarsvaoio, Iceland

## Abstract

**Background:**

Depression significantly impacts the overall well-being and quality of life in older adults, leading to reduced engagement in social and physical activities and an increased vulnerability to disability. Older individuals living alone may be more prone to have depressive feelings than those residing with a spouse or family members. This study aimed to investigate the association between living status and high depressive symptoms among Icelandic older adults.

**Methods:**

The analytical sample comprised 4,492 participants with a mean age of 76.3 years, of which 2,564 were women (57.1%), drawn from the Age, Gene/Environment Susceptibility-Reykjavik Study. Participants with diagnosed dementia and clinical depression were excluded from the analysis. Living arrangements were categorized as living alone (n=1,650, 36.7%) or with others. High depressive symptomatology (HDS) was defined as a Geriatric Depression Scale-15 score of 6 or higher (n=236, 5.3%). Logistic regression analysis was performed, adjusting for age, gender, education, body size, diabetes, hypertension, cholesterol, physical activity, and leisure activity.

**Results:**

After full adjustment, individuals living alone showed a significantly higher likelihood of experiencing HDS compared to those living with others (Beta = 1.46, 95% CI: 1.10 ~ 1.94, P <= 0.01).

**Conclusion:**

These findings emphasize the critical need to recognize potential mental health implications for older adults living alone, underscoring the imperative for targeted interventions and supportive healthcare systems. Addressing the unique challenges faced by solitary older adults is crucial for enhancing their mental well-being and overall quality of life.

